# An investigation of ^125^I seed permanent implantation for recurrent carcinoma in the head and neck after surgery and external beam radiotherapy

**DOI:** 10.1186/1477-7819-11-60

**Published:** 2013-03-08

**Authors:** Lihong Zhu, Yuliang Jiang, Junjie Wang, Weiqiang Ran, Huishu Yuan, Chen Liu, Ang Qu, Ruijie Yang

**Affiliations:** 1 Department of Radiation Oncology, Peking University 3rd Hospital, No. 49 Huayuan North road, Haidian district, Beijing, 100191, People’s Republic of China; 2 Department of Ultrasound, Peking University 3rd Hospital, No. 49 Huayuan North road, Haidian district, Beijing, 100191, People’s Republic of China; 3 Department of Radiology, Peking University 3rd Hospital, No. 49 Huayuan North road, Haidian district, Beijing, 100191, People’s Republic of China

**Keywords:** ^125^I seed, Head and neck carcinoma, Surgery, External beam radiotherapy

## Abstract

**Background:**

A preliminary assessment was conducted of the feasibility, efficacy, and morbidity of ^125^I seed implantation for recurrent head and neck carcinoma after surgery and external beam radiotherapy.

**Methods:**

Nineteen patients with recurrent head and neck carcinomas underwent ^125^I seed implantation under ultrasound or computed tomography guidance. The actuarial D90 of ^125^I seed implantation ranged from 90 to 160 Gy (median, 131 Gy). The follow-up period ranged from 3 to 44 months (median, 11 months).

**Results:**

The median local control was 24 months (95% confidence interval, 10.2 to 37.8). The one- year, two-year and three-year local controls were 73.3%, 27.5% and 27.5%, respectively, whereas the one-year, two-year and three-year survival rates were 53.0%, 18.2% and 18.2%, respectively, and the median survival was 13 months (95% confidence interval, 6.6 to 19.4). A total of 26.3% of patients (5/19) died of local recurrence and 21.1% of patients (4/19) died of metastases. One suffered from a grade 1 skin reaction.

**Conclusions:**

^125^I seed implantation is feasible and safe as a salvage treatment for patients with recurrent head and neck cancers. The high local control results and low morbidity merits further investigation.

## Background

The management of local and regional recurrence after surgery and radiotherapy in order to achieve a good outcome is often difficult. Survival in patients with recurrent head and neck carcinoma remains poor in spite of combined surgical resection and external beam radiation therapy (EBRT), with the major cause of death still being local and regional failure [1,2].

Many patients with local recurrence have had prior surgery and EBRT, and further EBRT is often difficult or impossible [3]. When a carcinoma recurs in a previously treated field, it presents very difficult problems for management. Salvage surgery is often technically feasible; however, the curative potential of surgery alone is poor in this condition. Unfortunately, local failure in these cases is at least 40% [4]. Re-EBRT has been used infrequently in the past decade because of previous high radiation dosage and concern regarding limited normal tissue tolerance. Nevertheless, local control rates of up to 50% and a five-year survival rate of 20% have been reported with re-EBRT [5-7]. The addition of concomitant chemotherapy to re-irradiation of recurrent head and neck carcinomas after previous full dose radiotherapy has achieved encouraging median survivals and long term disease-free survival [8-10].

Chemotherapy is widely used for palliation in patients with recurrent and unresectable head and neck carcinomas [11]. For locally advanced or recurrent head and neck cancer patients, temporary intraoperative high–dose-rate (HDR) brachytherapy combined with surgery may offer an alternative possibility, but the requirements are very high and the procedure is very complicated [12-14]. Interstitial implantation has also been used for salvage treatment of recurrent head and neck carcinoma, with a 40% to 60% local control rate and a 14% five-year survival rate [15-18]. Pulsed-dose rate (PDR) brachytherapy can offer an alternative re-irradiation possibility with less risk of severe morbidity [19]. In carefully selected patients, excellent local control rates of up to 80% with minimal side effects have been achieved [20,21]. Unfortunately, the size and the localization of most recurrences do not allow the optimal use of interstitial therapy.

Permanent implantation of ^125^I seeds into the tumor has the major advantages of delivering a high dose of irradiation to the tumor with a very sharp fall-off outside the implanted volume [22,23]. This concept is very important for head and neck cancers to maximize local control and minimize morbidity [24-26]. This article investigates the feasibility and efficacy of ^125^I seed implantation using computed tomography (CT) or ultrasound for guidance in the management of recurrent head and neck carcinomas and analyzes the local control and survival rates.

## Methods

### Patient information and selection

We retrospectively analyzed 19 patients (median age, 56 years; range, 19 to 86) who were treated with ^125^I seed implantation guided by ultrasound or CT in our institution for recurrent head and neck carcinoma after surgery and radiotherapy between January 2004 and December 2009. The criteria for eligibility were as follows: histologically proven recurrent head and neck carcinoma after surgery and radiotherapy with no evidence of distant metastasis; Karnofsky Performance Status (KPS) [27] of 60 or higher; and no major impairment of kidney, liver, or bone marrow function. The diameter of the mass is less than 9 cm. The patient characteristics are shown in Table 1.

**Table 1:** Characteristics of the patients (number = 19)

	**Number of patients**	**Percentage (%)**
Gender		
Male	11	57.9
Female	8	42.1
Median age	56(19 to 86)	
Primary tumor stage		
Stage I	1	5.3
Stage II	4	21.1
Stage III	7	36.8
Stage IV	7	36.8
Primary tumor		
Larynx	6	31.6
Soft tissue sarcoma	3	15.8
Hypopharynx carcinoma	2	10.5
Thyroid carcinoma	2	10.5
Cancer of nasal cavities	2	10.5
Nasopharynx carcinoma	1	5.3
Submandibular gland carcinoma	1	5.3
Maxillary sinus carcinoma	1	5.3
Merkel’s cells tumor of left ear	1	5.3
Tumor pathology		
Squamous cell carcinoma	9	47.4
Adenoid cystic carcinoma	2	10.5
Small cell carcinoma	1	5.3
Chondrosarcoma	1	5.3
Neurofibrosarcoma	1	5.3
Rhabdomyosarcoma	1	5.3
Medullary carcinoma	1	5.3
Papillary adenocarcinoma	1	5.3
Malignant melanoma	1	5.3
Merkel’s cells tumor	1	5.3
Previous surgery	19	100
One	12	63.2
Two	6	31.6
Three	1	5.2
Previous chemotherapy	5	26.3
Previous radiotherapy	19	100
One	12	63.1
Two	6	31.6
Three	1	5.3
Previous cumulative dose (Gy)		
≤50 Gy	3	15.8
50 to 100 Gy	13	68.4
>100 Gy	3	15.8
Median dose(Gy)	64(34 to 14)	

All patients had received radical surgery plus cervical lymphadenectomy. Twelve patients had received surgery once, six patients had received surgery twice, and one patient had received surgery three times. Twelve patients had recurrence after receiving a single course of EBRT, whereas six patients had recurrence after receiving two courses of EBRT. One patient had recurrence after three courses of EBRT. The total cumulative doses ranged from 34 to 145 Gy with 180 to 200 cGy per fraction (median total doses, 64 Gy). Five of the nineteen patients received a median of four cycle chemotherapy after EBRT (range, 1 to 6). Two patients experienced a grade 2 salivary gland reaction after EBRT. Three, two and two patients experienced a grade 1, grade 2 and grade 3 subcutaneous tissue reaction after EBRT, respectively. Three patients and one patient suffered from grade 1 and grade 2 skin reactions after EBRT, respectively. Two patients each suffered from grade 2 and grade 3 mucous reactions after EBRT, respectively. Seven patients had a recurrent carcinoma in the primary site and twelve patients in regional lymph nodes.

All patients had been interviewed by surgeons and radiation oncologists, and were considered not suitable for salvage surgery and EBRT, whereas others refused to undergo surgery and EBRT. The protocol was approved by a local ethics committee and written informed consent was obtained from the patients for publication of this report and any accompanying images. The title is “The study of dosimetry and quality control of image guided radioactive seed implantation treatment for recurrent head and neck cancer”.

### Treatment planning

All patients underwent a detailed tumor volume study using CT scans with a 5-mm thickness one to two weeks before seed implantation. The radiation oncologist outlined the planning target volume (PTV) on each transverse image. The tumor volume for the 19 patients varied from 1 to 270 cm^3^, with a median of 20 cm^3^. The D90 (the doses delivered to 90% of the target volume defined by CT using dose-volume histograms) of irradiation was determined. The total activity and the number of ^125^I seeds to be implanted in the target were obtained using the Three Dimensional Radiation Therapy Planning System (3D-TPS, Beijing Fei Tian Industries Inc., Beijing, China).

### Interstitial brachytherapy technique

Following the administration of adequate local anesthesia, fifteen patients underwent seed implantation under ultrasound guidance and four patients underwent seed implantation under CT guidance. After the direction of the needles was determination, the 18-gauge needles were punctured into the tumor by an interventional doctor, the needles kept in a parallel array with a 1.0 cm distance from each other. The direction of the needles has to be adjusted in order to avoid the large blood vessels and keep the needles 1 cm away from the organ at risk. ^125^I seeds were implanted using a Mick applicator, and the space between seeds was maintained at approximately 1.0 cm (center-to-center) after all needles had been implanted into the tumor. The needles were then removed. All patients received perioperative prophylactic antibiotics.

### Postimplant dosimetry evaluation

**Figure 1:**
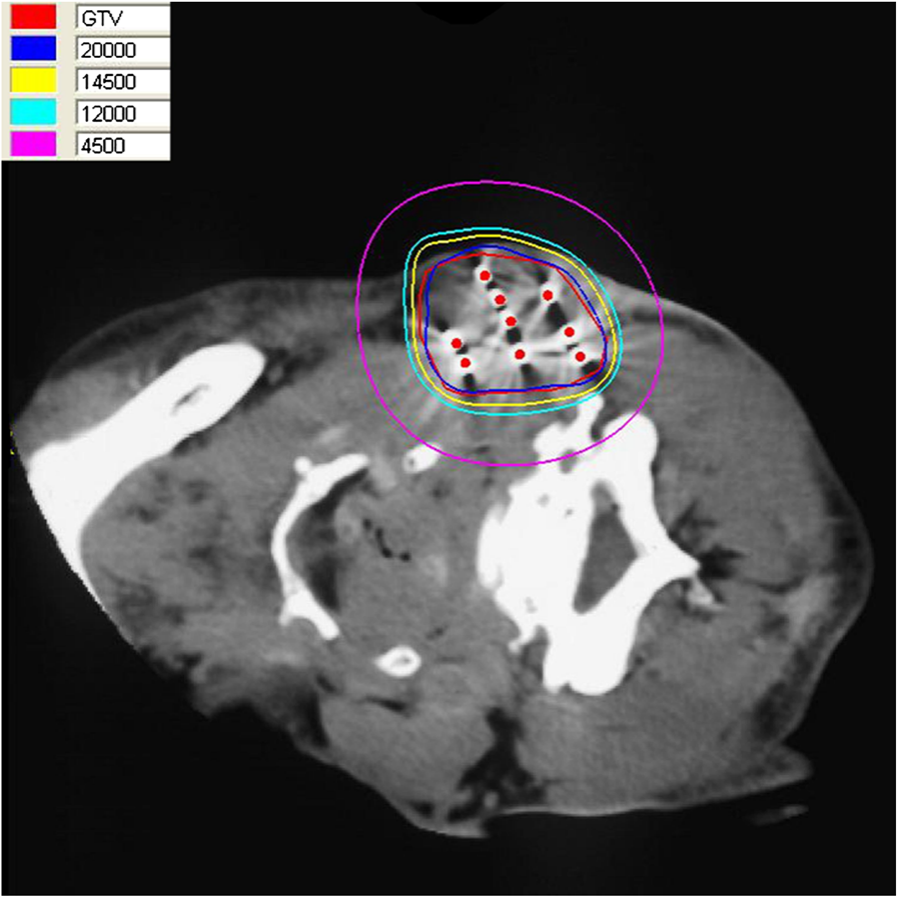
**The isodose curve distribution of tumor after seed implantation from CT scan.** The inner red curve represents tumor. The ellipses are iso-dose lines of 200, 145, 120 and 45 Gy from inside, respectively.

**Figure 2:**
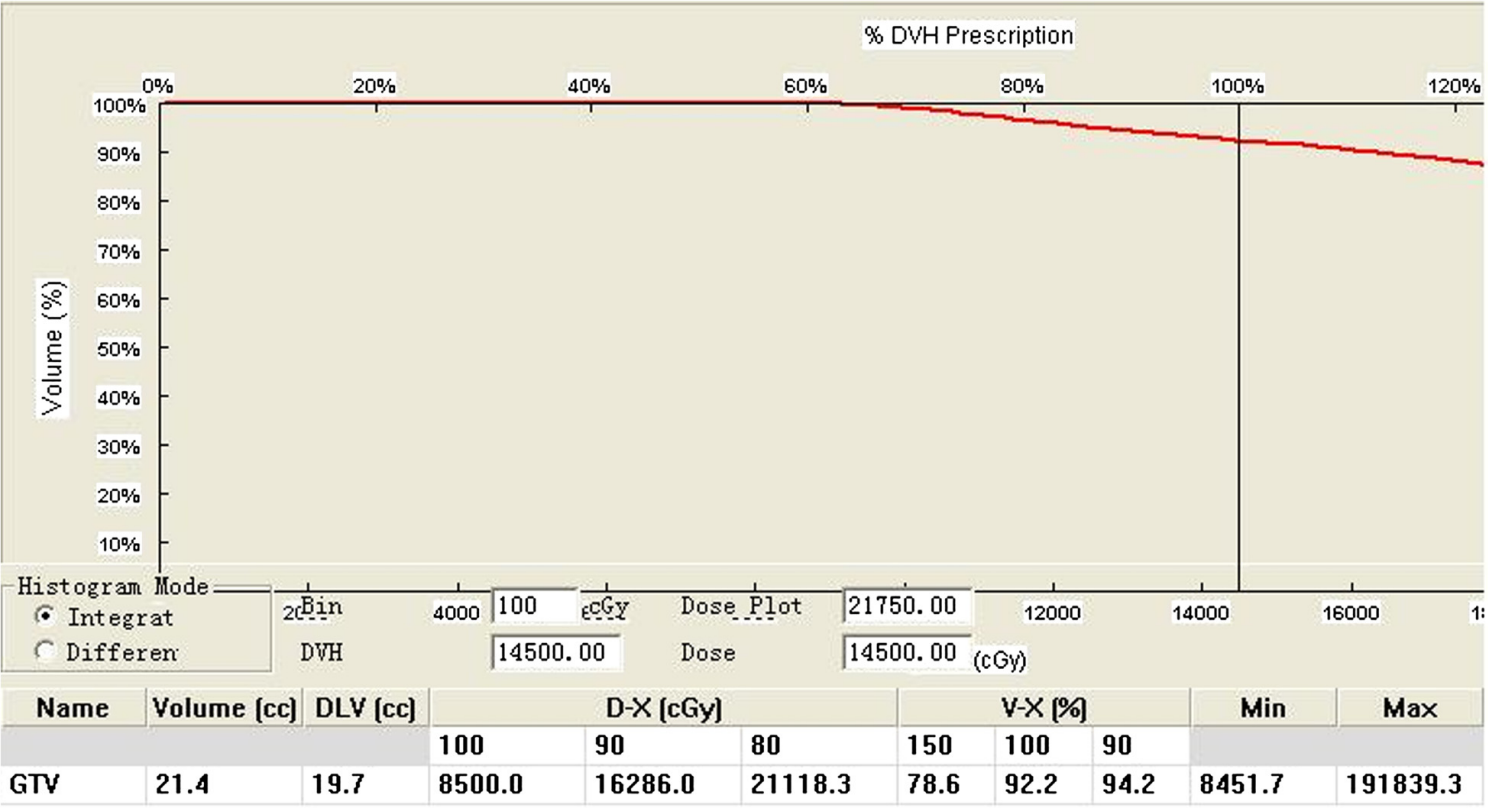
**The dose-volume histograms of gross tumor volume after seed implantation.** DVH: Dose volume histogram. The DVH is showing the target volume coverage by a certain dose level. DLV: This is the amount of the volume receiving the Defined Dose Level. D-X is defined as the minimum dose covering X% of the target volume. V-X is defined as the percent volume of the target receiving at least X% of the prescribed minimum peripheral dose (MPD).

Postoperative dosimetry was routinely performed for all patients. The implant dose was determined using three-dimensional seed identification and a 5 mm thickness CT scan immediately or 24 hours after seed implantation. The contored images and sources were entered into computerized treatment planning system software version 3.02 (Prowess – 3D, SSGI, Norwood, Massachusetts, USA , and a redundancy check was performed to prevent seed duplication. The median number of ^125^I seeds implanted was 29 (range, 3 to 78). The specific activity of the ^125^I seeds ranged from 0.40 to 0.80 mCi per seed, with a median of 0.67 mCi. The total amount of activity ranged from 1.8 to 46.02 mCi, with a median of 19.6 mCi. Over the period of total decay, the actuarial D90 was 90 to 160 Gy, with a median of 131 Gy (Figure 1 and Figure 2).

#### Follow-up

Tumor response was initially evaluated at four weeks and thereafter every two to three months. Disease status was assessed by physical examinations, liver function tests, and complete blood and platelet counts. The presence of disease progression was determined by means of imaging studies (CT scans and ultrasonography). The follow-up time was calculated from the date of seed implantation. The median follow-up was 11 months (range, 3 to 44 months). Complications were scored using the Radiation Therapy Oncology Group (RTOG)/European Organization for Research and Treatment of Cancer (EORTC) Late Radiation Morbidity Score [28].

#### Statistical methods

Survival time was calculated from the date of the seed implantation to the date of death or to the last follow-up. For calculation of survival, deaths due to any reason were scored as events. Local control (LC) was defined as lack of tumor progression of the implanted volume. Tumor responses were documented by CT and assessed using World Health Organization (WHO) criteria [29]. Overall survival curves and LC were generated using the Kaplan - Meier method and the Statistical Package for the Social Sciences (SPSS) 10.0 software.

## Results

### Local control

**Figure 3:**
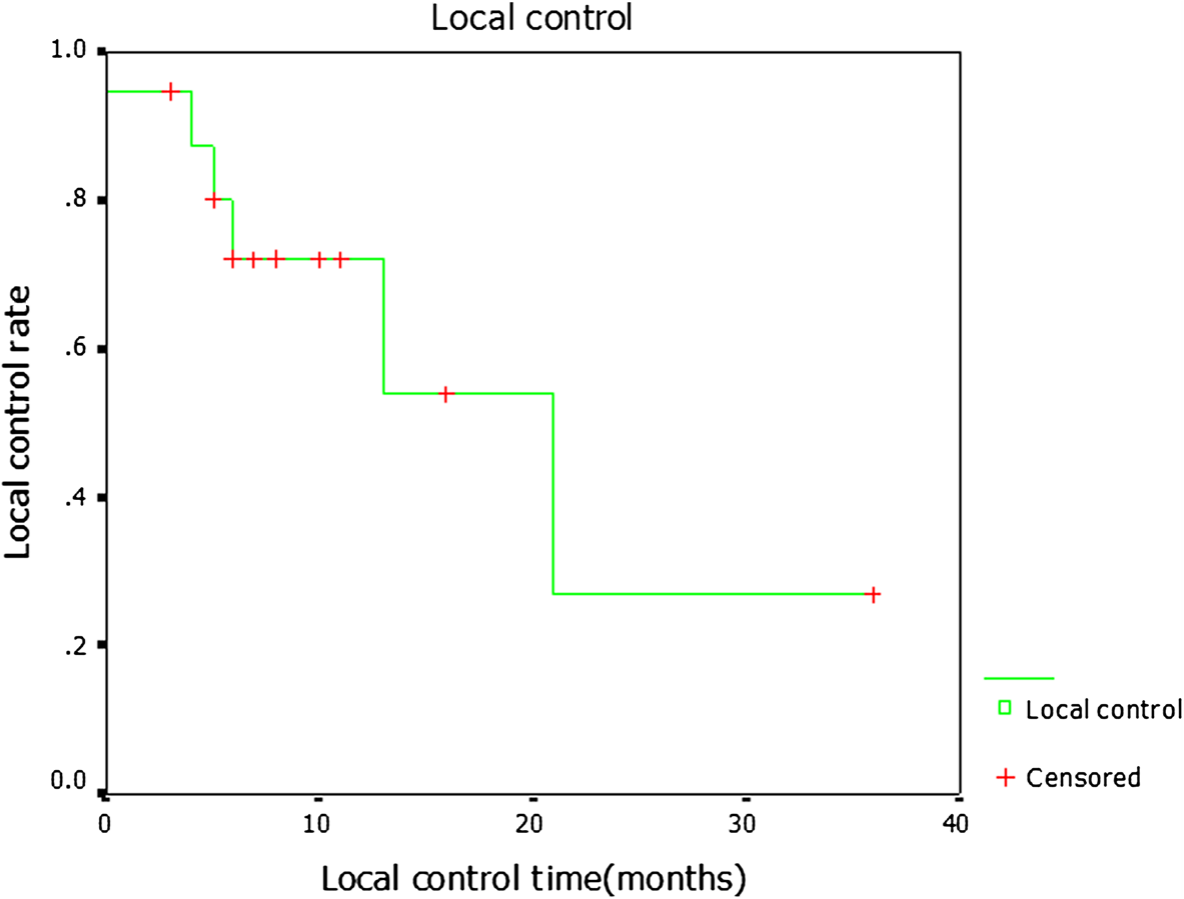
**Kaplan-Meier estimate data showing local control for all the patients after **^**125**^**I seed implantation.**

The overall response rate (complete response + partial response; CR + PR) for all nineteen patients was 73.7%, including two patients with CR (10.5%) and twelve patients with PR (63.2%). Four patients had stable disease (SD; 21.1%), and one had progressive disease (PD; 5.3%). The overall LC rate was 68.4% (13/19), with a median LC time of 24 months (95% CI: 10.2 to 37.8). The one-, two- and three- year LC rates were 73.3%, 27.5 % and 27.5%, respectively. A total of 26.3% patients (5/19) died of local recurrence. One patient with SD died from pneumonia three months after seed implantation, and one patient with PD died of pneumonia eight months after seed implantation (Figure 3).

### Survival

**Figure 4:**
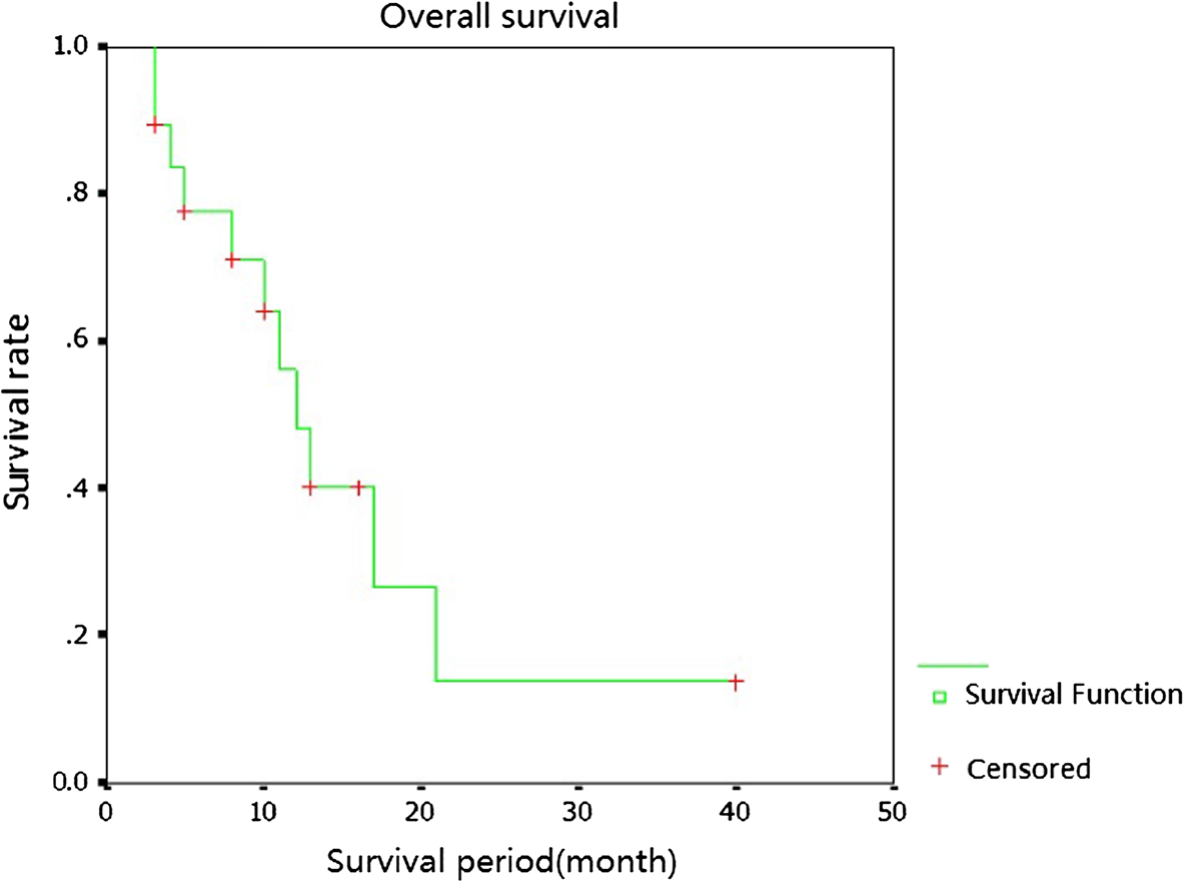
**Kaplan-Meier estimate data showing overall survival for all the patients after **^**125**^**I seed implantation.**

At the time of analysis, the median survival was 13 months (95% CI, 8.70 to 15.30 months). The one-, two- and three- year actuarial overall survivals were 53.0%, 18.2% and 18.2%, respectively. The cause of death in four patients (21.1%) who had died at the time of analysis was distant metastases (Figure 4). The patients who died with metastatic disease were all locally controlled.

### Complications

One patient suffered from a grade 1 skin reaction and another suffered from ulceration associated with tumor progression and died of local recurrence at 11 months after seed implantation. No soft tissue necrosis, neuropathy or carotid damages caused by irradiation were noted. Furthermore, no RTOG grade 4 or 5 complications were observed.

## Discussion

Seed-implant brachytherapy with image-guidance has been extensively performed in early stage prostate carcinoma treatment in recent years. The image-guidance brachytherapy improves seed deposition accuracy, and thus it delivers a sufficiently high dose to the tumor target, especially for recurrent carcinoma after EBRT. Additionally, because of the low energy of the radioactive sources of ^125^I, the doses to the surrounding normal tissues decrease very rapidly with distance, and are easily confined within the tumor target. Thus, seed implant brachytherapy provides superior high doses to the target mass and spares distant normal tissues.

Radioactive isotope permanent implantation in the management of head and neck cancer has been used for many decades. Radium and iridium-192 have been used for T1 and T2 patients with oral cavity and oropharynx carcinoma, the control rates of which are very high with functional results remaining very good [30-33]. Vikram *et al*. treated 124 patients with advanced recurrent head and neck cancer using ^125^I implants, 71% of whom underwent complete regression,18% showed more than 50% regression, and 11% showed no response. Overall, 64% of the patients remained controlled until their deaths. Only 9% of the patients survived for two years and 5.5% survived for five years. The rate of serious complications was 5.5% [34]. Goffinet *et al*. reported using permanent ^125^I seed implants as a surgical adjuvant in the management of advanced recurrent cancer of the head and neck; the majority of patients in his study had received prior treatment. Management involved a salvage operation combined with permanent implants using iodine-125 seeds. In this study, a 70% local control rate was achieved [35]. Park *et al*. reported on 35 patients with advanced recurrent squamous cell cancers of the head and neck. These patients were treated with surgical resection followed by adjuvant ^125^I seed implants. The decision for implantation was based upon either positive or close margins of resection after salvage operations. The determined five-year disease-free survival was 41% [36]. Ashamalla *et al*. reported on 94 patients who were treated with gold grain implants, in whom the total radiation dose ranged from 40 to 120 Gy, with a median of 80 Gy [37]. Complete LC was achieved in 33% of patients, and palliation was successfully accomplished in 76% of the cases. Cessation of bleeding occurred in 50% of the subjects, pain control was achieved in 88%, and 60% experienced relief from dysphagia. Our results are similar to these findings. Our results showed that the one–, two- and three- year control rates were 73.3%, 27.5% and 27.5%, respectively, with a median 24 months of local disease-free progression. The one–, two- and three-year actuarial overall survivals were 53.0%, 18.2% and 18.2%, respectively, with a median survival of 13 months.

Although it has been demonstrated that performing concomitant intraoperative HDR brachytherapy enhances actuarial survival and LC, local complications also have also been reported, varying from 11% to 56% [36]. Martinez *et al*. have reported an overall complication rate of 11% among 55 reconstructions performed in 48 patients [38]. Goffinet *et al*. reported such complications in approximately half of their patients. The main complications were skin ulceration and wound break-down [35]. Occasionally, patients experienced carotid rupture, which can be fatal. The key to preventing complications is a good implant technique and the liberal use of myocutaneous flaps for good coverage. Most interstitial brachytherapy is performed based on preoperative images and clinical presentation by placing the needles prior to treatment planning. The treatment plan is devised based on the achieved location of the needles in the operation. Unfortunately, the size and location of most recurrences do not allow the optimal use of interstitial therapy. The quality of an implant depends on the coverage of the PTV and the implant geometry, enabling a homogeneous dose distribution. It is very difficult to implant needles in parallel in tumor targets and to avoid the organ at risk. At the same time, the complications of intraoperative implantation of ^125^I seeds are high. It is difficult to define accurately the clinical target volume and the margin around the tumor under direct vision. Further, the seeds cannot be implanted in the defined volume, as any area left unimplanted will receive very little irradiation. Further, preplanning of seed needle implantations has a number of potential disadvantages. In particular, alterations in organ volume and shape between the time of the preplan and implant procedure, and the necessity of registering the pre-implant image with the actual patient position and set-up, may introduce inaccuracies in the implantation process. Krempien *et al*. reported the feasibility and accuracy of frameless image-guided interstitial needle implantation for 14 patients with locally recurrent head and neck cancers [39]. The results showed that the one- and two-year LC rates were 78% and 57%, respectively, and that the actuarial one- and two-year survival rates were 83% and 64%, respectively. Image guidance allows virtual planning and navigated implantation of brachytherapy needles with regard to optimized needle distribution and risk structures. We modified the implantation procedure by using CT or ultrasound guidance.

^125^I seed implantation with ultrasound or CT guidance has other advantages: 1)the implant technique is performed easily under local anesthesia without severe pain and discomfort during hospitalization; and 2) CT or ultrasound guidance reduces geographical miss, minimizes the radiation dose to the surrounding organs due to the sharp dose fall-off outside the implanted volume, and enhances sublethal damage repair, thereby protecting healthy organs from late tissue damage. We endeavored to improve local control and decrease sequelae by performing seed implantation under image guidance. The patients in our study were considered to have good performance status and a reasonable expectation of prolonged survival. We observed a very low rate of complications. One patient suffered from a grade 1 skin reaction, another patient suffered from an ulcer as a result of tumor progression and died of local recurrence 11 months after seed implantation. No bone, soft tissue necrosis or carotid artery damages were noted.

## Conclusion

CT or ultrasound-guided interstitial permanent ^125^I seed implantation is an effective re-irradiation modality for recurrent head and neck carcinomas after surgery and EBRT. It avoids the morbidity associated with further surgery or EBRT, achieves good local control, and the side effects are minimal. However, considering the small number of patients and the short follow-up period, a definite conclusion will require a larger number of patients and follow-up over a longer term.

## Abbreviations

CR: Complete response; CT: Computed tomography; DVH: Dose volume histogram; EBRT: External beam radiotherapy; EORTC: European Organization for Research and Treatment of Cancer; GTV: Gross tumor volume; HDR: High-dose rate; I: Iodine-125; LC: Local control; OS: Overall survival; PD: Progressive disease; PDR: Pulsed-dose-rate; PR: Partial response; PTV: Planning target volume; RTOG: Radiation Therapy Oncology Group; SD: Stable disease; TPS: Treatment planning system; WHO: World Health Organization

## Competing interests

The authors declare that they have no competing interests.

## Authors’ contributions

WJJ assisted with manuscript preparation. ZLH participated in the conception and design of the study and carried out the surgery. JYL helped with figure preparation. SQT participated in data analysis and interpretation. All authors read and approved the final manuscript.
